# Supplementary material for: Genetic Basis of Seedling Root Traits in Common Wheat (Triticum aestivum L.) Identified by Genome-Wide Linkage Mapping
Source: Plants (Basel). 2025 Feb 6;14(3):490. doi: 10.3390/plants14030490 (PMC11820154; doi:10.3390/plants14030490)
Supplement: Supplementary file 1 [file plants-14-00490-s001.zip › Table S1.pdf]

Table S1 The root system related traits of the Wp-072/Wp-119 RIL population

Line	RL (cm)	RS (mm^2)	RV (mm^3)	RW (g)	RT
R72/119-1	77.5	11.5	177.0	0.019	151.0
R72/119-2	54.4	6.7	145.0	0.020	92.0
R72/119-3	49.8	8.0	85.0	0.022	133.7
R72/119-4	81.3	12.4	345.0	0.033	231.8
R72/119-5	74.8	11.2	506.0	0.033	188.0
R72/119-6	64.7	8.8	1026.0	0.030	364.8
R72/119-7	36.7	5.5	107.0	0.025	175.5
R72/119-8	35.6	5.7	46.0	0.022	202.7
R72/119-9	57.8	10.1	204.0	0.024	177.5
R72/119-10	70.1	11.4	155.0	0.022	166.7
R72/119-11	106.7	15.7	589.0	0.035	274.0
R72/119-12	116.1	14.1	512.0	0.024	127.0
R72/119-13	45.6	6.2	32.0	0.019	83.3
R72/119-14	99.0	12.4	433.0	0.026	200.3
R72/119-15	118.3	13.2		0.031	303.3
R72/119-16	144.1	13.4	597.0	0.032	251.0
R72/119-17	117.9	16.6	656.0	0.033	262.8
R72/119-18	81.8	11.9	204.0	0.024	145.2
R72/119-19	71.5	12.3	251.0	0.026	182.2
R72/119-20	65.6	9.3	196.0	0.021	142.3
R72/119-21	123.7	11.2	871.0	0.033	289.3
R72/119-22	146.3	15.6	661.0	0.032	266.0
R72/119-23	51.6	8.7	341.0	0.022	210.7
R72/119-24	56.9	7.8	428.0	0.034	238.7
R72/119-25	63.2	9.4	100.0	0.020	121.2
R72/119-26	97.2	12.4	985.0	0.033	368.3
R72/119-27	59.8	10.5	341.0	0.036	334.2
R72/119-28	117.0	14.9	274.0	0.030	239.8
R72/119-29	116.9	13.5		0.031	
R72/119-30	71.0	7.8	970.0	0.034	
R72/119-31	81.1	10.5		0.037	
R72/119-32	62.9	8.4	409.0	0.037	268.8
R72/119-33	66.9	7.5	270.0	0.027	208.0
R72/119-34	61.4	8.8	241.0	0.023	198.3
R72/119-35	57.0	8.1	213.0	0.035	274.5
R72/119-36	82.9	8.8	371.0	0.026	174.0
R72/119-37	95.3	12.2	589.0	0.040	381.5
R72/119-38	133.6	16.7	1027.0	0.046	389.3
R72/119-39	135.6	12.0	539.0	0.037	208.2
R72/119-40	80.3	10.3	405.0	0.033	284.5
R72/119-41	130.0	12.7	713.0	0.029	245.8
R72/119-42	69.7	9.6	416.0	0.035	196.7

R72/119-43	69.5	12.1	169.0	0.031	204.8
R72/119-44	107.6	11.8	468.0	0.028	207.8
R72/119-45	118.1	15.6	391.0	0.033	211.2
R72/119-46	93.0	10.4	391.0	0.022	168.7
R72/119-47	53.2	9.0	281.0	0.022	150.3
R72/119-48	115.5	11.0	662.0	0.037	260.7
R72/119-49	79.0	12.9	528.0	0.042	259.3
R72/119-50	112.8	12.6	479.0	0.036	263.2
R72/119-51	90.9	10.9	426.0	0.025	222.0
R72/119-52	123.8	15.5	889.0	0.034	337.5
R72/119-53	71.7	8.2	408.0	0.033	334.7
R72/119-54	87.6	14.3	354.0	0.034	238.0
R72/119-55	63.6	10.3	136.0	0.027	196.0
R72/119-56	103.7	12.3	308.0	0.026	267.5
R72/119-57	74.4	10.3	81.0	0.029	197.0
R72/119-58	59.1	9.2	231.0	0.034	321.7
R72/119-59	50.0	7.6	376.0	0.027	270.8
R72/119-60	77.2	12.0	599.0	0.038	371.7
R72/119-61	112.1	14.3		0.033	294.5
R72/119-62	110.2	16.5	372.0	0.034	
R72/119-63	128.4	14.5	308.0	0.035	277.7
R72/119-64	89.6	12.3	920.0	0.032	383.8
R72/119-65	121.7	11.7	518.0	0.031	292.0
R72/119-66	110.0	12.4	492.0	0.027	254.3
R72/119-67	46.3	6.8	448.0	0.025	223.5
R72/119-68	98.1	9.7	115.0	0.028	150.3
R72/119-69	56.2	11.6	511.0	0.033	227.2
R72/119-70	63.1	9.7	148.0	0.031	234.0
R72/119-71	87.8	8.7	333.0	0.038	238.3
R72/119-72	36.6	6.3	709.0	0.027	305.8
R72/119-73	85.1	10.2	142.0	0.031	149.7
R72/119-74	101.5	11.2	422.0	0.036	282.3
R72/119-75	84.8	10.8	692.0	0.028	289.8
R72/119-76	90.2	12.8	666.0	0.028	244.5
R72/119-77	104.6	10.4	518.0	0.025	224.7
R72/119-78	64.9	6.8	711.0	0.025	206.8
R72/119-79	51.0	7.2	168.0	0.027	126.3
R72/119-80	77.5	10.6	234.0	0.029	158.2
R72/119-81	89.3	14.2	381.0	0.028	234.5
R72/119-82	54.3	7.5	460.0	0.026	264.0
R72/119-83	101.6	11.0	333.0	0.031	215.0
R72/119-84	57.4	8.7	661.0	0.024	237.3
R72/119-85	49.5	8.1	153.0	0.034	179.2
R72/119-86	112.5	10.8	276.0	0.022	342.7

R72/119-87	59.1	7.5	843.0	0.033	330.0
R72/119-88	110.3	11.7	400.0	0.039	339.7
R72/119-89	86.6	11.4	499.0	0.025	284.7
R72/119-90	84.6	9.6	134.0	0.027	124.8
R72/119-91	72.6	8.0	341.0	0.023	165.0
R72/119-92	123.8	13.3	113.0	0.028	202.2
R72/119-93	104.1	16.4	445.0	0.028	211.7
R72/119-94	59.0	9.6	443.0	0.030	337.2
R72/119-95	136.4	15.5	138.0	0.029	136.0
R72/119-96	78.9	9.6	301.0	0.026	242.8
R72/119-97	137.8	16.9	333.0	0.039	161.0
R72/119-98	94.9	9.8	904.0	0.026	311.8
R72/119-99	62.1	6.5	443.0	0.020	169.2
R72/119-100	66.6	9.4	76.0	0.026	119.8
R72/119-101	85.6	9.6	163.0	0.022	121.3
R72/119-102	95.3	11.8	409.0	0.033	168.8
R72/119-103	71.1	12.6	322.0	0.023	
R72/119-104	37.3	4.0	539.0	0.029	220.7
R72/119-105	86.7	10.9	267.0	0.031	
R72/119-106	84.4	10.9	342.0	0.030	
R72/119-107	62.2	9.5	237.0	0.031	
R72/119-108	88.2	11.7	155.0	0.025	198.0
R72/119-109	98.0	11.2	350.0	0.023	143.7
R72/119-110	126.3	13.8	294.0	0.037	
R72/119-111	86.4	11.5	798.0	0.032	
R72/119-112	75.1	13.1	124.0	0.024	208.0
R72/119-113	73.1	9.2	84.0	0.026	115.0
R72/119-114	108.7	12.3	138.0	0.031	114.2
R72/119-115	66.2	11.1	731.0	0.041	276.0
R72/119-116	70.9	12.4	465.0	0.028	207.7
R72/119-117	78.3	7.4	345.0	0.026	240.0
R72/119-118	72.7	11.3	479.0	0.041	198.5
R72/119-119	133.3	17.5	235.0	0.034	291.7
R72/119-120	144.2	13.1	944.0	0.028	256.8
R72/119-121	70.6	10.8	587.0	0.030	203.0
R72/119-122	133.1	11.9	130.0	0.039	173.0
R72/119-123	91.2	12.3	772.0	0.034	282.3
R72/119-124	35.1	8.6	206.0	0.026	240.2
R72/119-125	95.1	9.1	529.0	0.037	215.8
R72/119-126	67.7	11.1	475.0	0.028	204.3
R72/119-127	48.8	11.5	195.0	0.027	219.0
R72/119-128	119.6	11.0	101.0	0.037	155.3
R72/119-129	94.4	8.7	635.0	0.032	330.3
R72/119-130	91.7	10.8	597.0	0.029	276.2

R72/119-131	76.6	13.4	556.0	0.031	180.0
R72/119-132	80.5	8.6	592.0	0.027	222.0
R72/119-133	90.4	12.8	537.0		
R72/119-134	94.6	12.4	491.0	0.029	251.7
R72/119-135	32.5	5.3	470.0	0.019	197.8
R72/119-136	51.7	7.7	129.0	0.015	109.5
R72/119-137	131.5	12.1	46.0	0.027	57.8
R72/119-138	40.2	10.4	590.0	0.021	167.0
R72/119-139	64.1	11.1	104.0	0.027	143.2
R72/119-140	90.1	17.4	189.0	0.033	222.5
R72/119-141	109.6	14.6	370.0	0.032	257.2
R72/119-142	145.3	11.6	323.0	0.032	185.5
R72/119-143	114.2	13.6	486.0	0.028	276.8
R72/119-144	25.4	5.8	411.0	0.017	199.3
R72/119-145	39.9	6.2	146.0	0.026	129.7
R72/119-146	100.8	9.3	257.0	0.033	153.5
R72/119-147	70.8	10.4	472.0	0.021	197.3
R72/119-148	53.1	7.4	187.0	0.027	139.5
R72/119-149	68.6	12.1	239.0	0.024	208.0
R72/119-150	40.4	6.4	237.0	0.025	140.2
R72/119-151	46.8	12.6	260.0	0.027	164.2
R72/119-152	51.9	7.2	208.0	0.035	220.0
R72/119-153	89.3	8.9	220.0	0.026	160.2
R72/119-154	77.7	16.5	674.0	0.028	217.3
R72/119-155	59.6	11.7	342.0	0.021	208.0
R72/119-156	28.7	7.2	267.0	0.027	117.2
R72/119-157	36.1	7.7	179.0	0.038	154.8
R72/119-158	64.3	8.6	219.0	0.033	227.2
R72/119-159	82.5	15.4	466.0	0.035	244.8
R72/119-160	50.7	8.2	770.0	0.029	253.7
R72/119-161	40.0	8.8	280.0	0.033	188.8
R72/119-162	46.6	10.4	338.0	0.023	343.2
R72/119-163	27.5	5.4	380.0	0.026	140.5
R72/119-164	42.8	9.6	141.0	0.035	274.2
R72/119-165	67.9	16.0	354.0	0.028	297.0
R72/119-166	86.7	10.3	340.0	0.034	171.0
R72/119-167	60.4	8.6	708.0	0.030	271.5
R72/119-168	67.6	8.0	394.0	0.036	189.5
R72/119-169	62.4	7.6	249.0	0.026	203.0
R72/119-170	65.9	11.9	220.0	0.028	255.5
R72/119-171	54.9	8.6	264.0	0.030	241.5
R72/119-172	33.4	8.8	225.0	0.037	112.2
R72/119-173	82.2	9.4	164.0	0.036	171.7
R72/119-174	42.0	8.4	531.0	0.030	199.2

R72/119-175	69.9	8.3	276.0	0.024	187.3
R72/119-176	101.7	13.1	334.0	0.037	184.8
R72/119-177	83.2	15.0	438.0	0.034	262.0
R72/119-178	34.4	6.0	451.0	0.032	279.2
R72/119-179	57.1	7.7	324.0	0.041	209.3
R72/119-180	44.2	7.6	444.0	0.029	348.2
R72/119-181	66.7	14.3	269.0	0.039	202.8
R72/119-182	70.7	11.1	337.0	0.024	210.0
R72/119-183	41.1	9.4	398.0	0.029	180.2
R72/119-184	71.0	8.0	153.0	0.029	209.8
R72/119-185	63.2	7.9	516.0	0.035	139.2
R72/119-186	51.0	7.5	480.0	0.033	244.2
R72/119-187	71.8	12.8	428.0	0.039	269.5
R72/119-188	65.9	8.2	637.0	0.028	310.8
R72/119-189	73.1	9.3	311.0	0.037	213.0
R72/119-190	87.0	10.1	260.0	0.025	192.7
R72/119-191	49.0	8.4	402.0	0.023	147.8
R72/119-192	59.1	7.8	225.0	0.022	131.3
R72/119-193	40.2	8.5	193.0	0.029	116.3
R72/119-194	41.8	6.5	176.0	0.037	157.3
R72/119-195	57.3	8.6	269.0	0.024	207.2
R72/119-196	34.5	6.5	369.0	0.028	166.5
R72/119-197	41.5	8.8	55.0	0.031	97.7
R72/119-198	69.9	10.7	150.0	0.034	149.5
R72/119-199	66.8	13.8	391.0	0.042	196.8
R72/119-200	44.9	7.7	459.0	0.025	224.5
R72/119-201	42.5	8.3	179.0	0.043	139.7
R72/119-202	47.9	10.3	341.0	0.033	196.7
R72/119-203	64.3	10.5	346.0	0.041	187.3
R72/119-204	28.5	8.9	246.0	0.032	163.0
R72/119-205	47.8	9.7	91.0	0.032	138.3
R72/119-206	53.9	9.1	192.0	0.030	118.0
R72/119-207	54.9	11.0	252.0	0.034	176.8
R72/119-208	79.4	12.7	258.0	0.042	176.8
R72/119-209	57.5	12.6	326.0	0.027	274.0
R72/119-210	43.1	5.3	310.0	0.045	144.0
R72/119-211	61.4	8.6	263.0	0.034	301.5
R72/119-212	29.6	6.2	536.0	0.024	281.0
R72/119-213	68.9	11.3	126.0	0.040	119.2
R72/119-214	50.7	10.6	410.0	0.022	253.7
R72/119-215	27.1	5.9	195.0	0.024	156.3
R72/119-216	24.8	6.6	125.0	0.019	137.0
R72/119-217	30.5	7.3	22.0	0.026	72.8
R72/119-218	51.9	7.6	151.0	0.041	112.3

R72/119-219	48.4	6.5	285.0	0.028	153.5
R72/119-220	55.6	10.9	151.0	0.036	194.2
R72/119-221	47.6	8.8	413.0	0.035	232.7
R72/119-222	56.3	14.2	377.0	0.042	248.5
R72/119-223	60.3	6.9	766.0	0.035	249.2
R72/119-224	85.5	11.7	320.0	0.031	240.8
R72/119-225	48.9	9.3	371.0	0.020	131.2
R72/119-226	44.9	9.6	153.0	0.036	175.2
R72/119-227	42.9	7.6	118.0	0.044	208.7
R72/119-228	94.5	11.3	253.0	0.039	184.7
R72/119-229	118.3	13.4	375.0	0.042	249.0
R72/119-230	70.9	10.9	558.0	0.041	254.3
R72/119-231	35.9	6.9	397.0	0.027	284.5
R72/119-232	79.8	7.3	261.0	0.036	210.2
R72/119-233	52.1	13.8	497.0	0.036	246.7
R72/119-234	43.0	8.2	644.0	0.030	236.5
R72/119-235	48.6	9.6	93.0	0.038	103.8
R72/119-236	57.1	10.1	435.0	0.030	220.7
R72/119-237	59.4	8.8	495.0	0.027	183.0
R72/119-238	65.1	10.3	454.0	0.031	269.2
R72/119-239	61.2	8.5	462.0	0.026	249.7
R72/119-240	44.2	6.9	282.0	0.035	195.3
R72/119-241	78.0	8.4	238.0	0.034	251.0
R72/119-242	101.8	10.9	321.0	0.029	211.7
R72/119-243	37.3	6.0	689.0	0.030	272.7

RL: root length; RA: root surface area; RV: root volume; RT: of root tips; RW: root dry weight.
