# Supplementary material for: Genetic Basis of Seedling Root Traits in Common Wheat (Triticum aestivum L.) Identified by Genome-Wide Linkage Mapping
Source: Plants (Basel). 2025 Feb 6;14(3):490. doi: 10.3390/plants14030490 (PMC11820154; doi:10.3390/plants14030490)
Supplement: Supplementary file 1 [file plants-14-00490-s001.zip › Table S2.pdf]

Table S2 The correlation analysis for the root system related traits in the Wp-072/Wp-119 RIL population

	RL	RA	RV	RW
RA	0.713**			
RV	0.273*	0.184		
RW	0.190	0.235*	0.120	
RT	0.256*	0.252*	0.678**	0.251*

RL: root length; RA: root surface area; RV: root volume; RT: root tips; RW: root dry weight.
