# Supplementary material for: Genetic Basis of Seedling Root Traits in Common Wheat (Triticum aestivum L.) Identified by Genome-Wide Linkage Mapping
Source: Plants (Basel). 2025 Feb 6;14(3):490. doi: 10.3390/plants14030490 (PMC11820154; doi:10.3390/plants14030490)
Supplement: Supplementary file 1 [file plants-14-00490-s001.zip › Table S3.pdf]

**Table S3** The genotype and RSA related traits for the 149 wheat accessions by developed KASP markers

| Name | Origin | RL (cm) | RS ($mm^2$) | RW (g) | *KASP-RL-1DL* | *KASP-RS-1D* | *KASP-RS-2DL* | *KASP-RW-2AS* | *KASP-RW-2DL* |
|---|---|---|---|---|---|---|---|---|---|
| CHANG6990 | Cultivar | 67.6 | 7.8 | 0.039 | CC | CC | AA | AA | GG |
| CUNMAI218 | Cultivar | 143.8 | 16.2 | 0.039 | CC | AA | GG | AA | GG |
| CUNMAI25 | Cultivar | 44.8 | 9.9 | 0.037 | CC | CC | AA | AA | GG |
| FANMAI26 | Cultivar | 70.8 | 10.6 | 0.023 | TC | CC | GG | GG | AA |
| FANMAI28 | Cultivar | 52.4 | 14.2 | 0.030 | TC | AA | GG | GG | GG |
| FANMAI30 | Cultivar | 132.5 | 15.0 | 0.046 | CC | AA | GG | AA | GG |
| FANMAI31 | Cultivar | 161.4 | 15.0 | 0.036 | CC | AA | GG | AA | GG |
| HANGMAI106 | Cultivar | 119.6 | 17.6 | 0.039 | CC | AA | GG | AA | AA |
| HANGMAI917 | Cultivar | 101.8 | 12.2 | 0.028 | TT | AA | GG | AA | GG |
| HUAIHE15076 | Cultivar | 62.3 | 12.3 | 0.040 | CC | AA | GG | AA | GG |
| HUAIHE15173 | Cultivar | 90.8 | 11.7 | 0.041 | CC | CC | GG | AA | GG |
| HUAIHE16174 | Cultivar | 47.6 | 9.3 | 0.048 | TT | AA | AA | AA | AA |
| HUAIHE19108 | Cultivar | 72.0 | 9.6 | 0.037 | TT | CC | AA | AA | GG |
| HUAIHE20224 | Cultivar | 47.0 | 9.4 | 0.034 | TT | TC | GG | AA | GG |

| | | | | | | | | | |
|---|---|---|---|---|---|---|---|---|---|
| HUAIHE21126 | Cultivar | 95.8 | 13.2 | 0.034 | TC | CC | GG | AA | GG |
| HUAIMAI2118 | Cultivar | 125.5 | 16.0 | 0.037 | TT | AA | GG | GG | AA |
| HUAIMAI33 | Cultivar | 132.0 | 18.6 | 0.037 | CC | AA | AA | AA | AA |
| HUAIMAI404 | Cultivar | 89.4 | 8.2 | 0.040 | CC | CC | AA | AA | AA |
| HUAIMAI606 | Cultivar | 95.3 | 11.4 | 0.035 | CC | CC | GG | AA | AA |
| HUAIMAI701 | Cultivar | 79.3 | 11.7 | 0.024 | CC | CC | AA | AA | GG |
| WP-072 | Cultivar | 27.8 | 7.4 | 0.021 | CC | CC | AA | GG | GG |
| JIMAI40 | Cultivar | 68.8 | 9.9 | 0.026 | CC | AA | AA | AA | GG |
| JIMAI44 | Cultivar | 84.1 | 14.6 | 0.026 | CC | AA | GG | AA | GG |
| JIMAI55 | Cultivar | 66.8 | 13.1 | 0.024 | TT | AA | AA | GG | GG |
| KENONG2009 | Cultivar | 61.5 | 9.7 | 0.034 | TT | CC | GG | GG | AA |
| LANTIAN15 | Cultivar | 51.8 | 7.6 | 0.028 | CC | AA | GG | AA | GG |
| LANTIAN26 | Cultivar | 63.7 | 8.8 | 0.038 | CC | CC | GG | GG | GG |
| LIANGXING66 | Cultivar | 72.1 | 11.7 | 0.046 | CC | CC | AA | AA | AA |
| LIANGXING99 | Cultivar | 67.6 | 9.6 | 0.034 | CC | AA | GG | GG | AA |
| LONGJIAN115 | Cultivar | 104.2 | 11.7 | 0.025 | CC | CC | AA | GG | GG |
| LONGJIAN386 | Cultivar | 121.8 | 13.8 | 0.034 | CC | CC | GG | AA | GG |

| | | | | | | | | |
|---|---|---|---|---|---|---|---|---|
| LUMAI23 | Cultivar | 44.7 | 6.9 | 0.029 | CC | CC | GG | GG | GG |
| LUMAI6HAO | Cultivar | 149.7 | 18.7 | 0.051 | CC | AA | GG | AA | AA |
| LUMAI7HAO | Cultivar | 106.5 | 10.2 | 0.042 | TT | AA | AA | AA | AA |
| LUNXUAN136 | Cultivar | 79.5 | 8.8 | 0.038 | CC | CC | GG | AA | GG |
| LUNXUAN139 | Cultivar | 73.5 | 10.4 | 0.024 | TT | TC | GG | GG | AA |
| LUNXUAN147 | Cultivar | 130.9 | 15.1 | 0.034 | CC | AA | GG | AA | GG |
| LUNXUAN67 | Cultivar | 38.5 | 6.7 | 0.036 | TT | CC | AA | GG | AG |
| LUOFENG2419 | Cultivar | 97.1 | 11.6 | 0.038 | CC | CC | AA | GG | GG |
| LUOFENG7011 | Cultivar | 80.4 | 14.3 | 0.044 | TT | AA | GG | AA | GG |
| LUOHAN2HAO | Cultivar | 52.1 | 11.6 | 0.025 | TT | CC | GG | GG | GG |
| LUOMAI1137 | Cultivar | 46.0 | 10.6 | 0.033 | CC | CC | GG | GG | GG |
| LUOMAI27 | Cultivar | 64.1 | 9.6 | 0.027 | CC | AA | GG | GG | GG |
| LUOMAI33 | Cultivar | 79.2 | 12.4 | 0.027 | TT | CC | AA | GG | GG |
| LUOMAI38 | Cultivar | 41.8 | 4.5 | 0.033 | CC | CC | GG | AA | GG |
| LUOMAI42 | Cultivar | 93.2 | 16.8 | 0.039 | CC | AA | GG | AA | AG |
| LUOMAI49 | Cultivar | 98.3 | 9.8 | 0.042 | TT | CC | GG | GG | AA |
| LUOMAI56 | Cultivar | 74.6 | 10.6 | 0.029 | CC | CC | AA | AA | GG |

| | | | | | | | | | |
|---|---|---|---|---|---|---|---|---|---|
| LUOMAI69 | Cultivar | 106.7 | 13.3 | 0.037 | TT | AA | AG | GG | AA |
| LUOMAI896 | Cultivar | 131.1 | 16.7 | 0.033 | CC | AA | GG | GG | GG |
| LUYAN148 | Cultivar | 71.2 | 11.5 | 0.031 | TT | CC | GG | AA | GG |
| LUYAN987 | Cultivar | 68.6 | 9.5 | 0.029 | TT | CC | AA | GG | GG |
| LUYUAN502 | Cultivar | 50.3 | 10.7 | 0.041 | TT | CC | AA | AA | GG |
| MALAN1 | Cultivar | 100.0 | 15.9 | 0.031 | TT | CC | AA | GG | GG |
| NONGDA759 | Cultivar | 126.0 | 12.1 | 0.025 | CC | TC | GG | AA | AA |
| PUBING01 | Cultivar | 122.8 | 16.3 | 0.036 | CC | CC | AA | GG | GG |
| PUBING03 | Cultivar | 37.4 | 9.9 | 0.042 | NN | CC | AG | GG | AG |
| PUBING300 | Cultivar | 98.2 | 16.0 | 0.038 | CC | CC | GG | AA | GG |
| SHANGMAI178 | Cultivar | 113.9 | 14.6 | 0.041 | TT | AA | GG | AA | AA |
| SHANGMAI187 | Cultivar | 91.1 | 13.8 | 0.037 | CC | AA | AA | GG | GG |
| SHANGMAI189 | Cultivar | 123.6 | 13.1 | 0.044 | TT | AA | GG | AA | AA |
| SHANGMAI206 | Cultivar | 101.0 | 19.5 | 0.037 | TT | AA | NN | GG | GG |
| SHANNONG24 | Cultivar | 66.1 | 10.8 | 0.033 | CC | CC | AA | AA | AA |
| SHI4185 | Cultivar | 48.2 | 9.2 | 0.034 | TT | AA | GG | AA | GG |
| WANKE2809 | Cultivar | 91.6 | 13.3 | 0.027 | CC | CC | GG | GG | GG |

| | | | | | | | | | |
|---|---|---|---|---|---|---|---|---|---|
| WANKE421 | Cultivar | 45.2 | 7.1 | 0.028 | CC | AA | GG | GG | GG |
| WANKE505 | Cultivar | 31.9 | 10.0 | 0.036 | TT | CC | GG | AA | GG |
| WANKE800 | Cultivar | 62.9 | 13.0 | 0.037 | TT | AA | GG | GG | GG |
| WANMAI38 | Cultivar | 64.3 | 9.8 | 0.027 | CC | CC | GG | GG | GG |
| WEILONG169 | Cultivar | 33.1 | 6.9 | 0.027 | CC | CC | AA | AA | GG |
| XINDONG52 | Cultivar | 162.7 | 13.0 | 0.036 | TT | AA | AA | GG | AA |
| XINMAI26 | Cultivar | 138.6 | 12.6 | 0.037 | TC | CC | GG | GG | AG |
| XINMAI38 | Cultivar | 113.7 | 12.5 | 0.040 | CC | CC | GG | AG | AA |
| XINMAI45 | Cultivar | 87.0 | 18.5 | 0.031 | TT | AA | GG | AA | GG |
| XINONG1155 | Cultivar | 59.6 | 10.1 | 0.024 | CC | TC | GG | GG | GG |
| XINONG135 | Cultivar | 47.7 | 9.4 | 0.034 | TT | CC | AG | GG | GG |
| XINONG1522 | Cultivar | 78.1 | 10.7 | 0.040 | CC | AA | GG | GG | GG |
| XINONG1668 | Cultivar | 106.0 | 13.9 | 0.032 | CC | AA | GG | GG | GG |
| XINONG20 | Cultivar | 105.7 | 9.7 | 0.036 | CC | CC | AA | AA | AA |
| XINONG222 | Cultivar | 60.9 | 7.5 | 0.022 | NN | CC | AA | GG | GG |
| XINONG306 | Cultivar | 98.8 | 13.1 | 0.028 | TT | CC | GG | GG | GG |
| XINONG362 | Cultivar | 69.9 | 8.5 | 0.029 | TT | CC | AA | GG | AA |

| | | | | | | | | | |
|---|---|---|---|---|---|---|---|---|---|
| XINONG511 | Cultivar | 136.3 | 13.1 | 0.034 | CC | AA | GG | GG | GG |
| XINONG66 | Cultivar | 54.7 | 10.5 | 0.022 | CC | CC | AA | GG | GG |
| XINONG733 | Cultivar | 75.7 | 8.9 | 0.040 | CC | AA | AA | GG | AA |
| XINONG840 | Cultivar | 45.0 | 11.6 | 0.024 | TT | AA | GG | GG | GG |
| XINONG969 | Cultivar | 85.8 | 15.0 | 0.035 | TC | CC | AA | AA | GG |
| XINONG979 | Cultivar | 75.8 | 12.5 | 0.032 | CC | CC | AA | AA | GG |
| XUMAI15019 | Cultivar | 70.7 | 8.9 | 0.039 | TT | CC | AA | AA | GG |
| XUMAI15194 | Cultivar | 89.9 | 11.6 | 0.037 | CC | CC | AA | AA | AA |
| XUMAI36 | Cultivar | 106.3 | 11.0 | 0.030 | TT | CC | GG | GG | GG |
| YANGMAI25 | Cultivar | 63.0 | 15.8 | 0.047 | CC | TC | GG | GG | GG |
| YANNONG1212 | Cultivar | 105.8 | 12.7 | 0.044 | CC | AA | AA | AA | GG |
| YANNONG15 | Cultivar | 116.6 | 18.4 | 0.031 | CC | AA | GG | GG | AA |
| YANNONG187 | Cultivar | 106.7 | 13.7 | 0.045 | TT | CC | AA | AA | GG |
| YANNONG999 | Cultivar | 38.7 | 7.3 | 0.031 | TT | AA | GG | GG | GG |
| YUMAI18 | Cultivar | 151.9 | 13.5 | 0.042 | CC | CC | GG | AA | GG |
| YUMAI34 | Cultivar | 64.0 | 11.4 | 0.034 | TT | CC | GG | GG | GG |
| YUMAI49 | Cultivar | 59.4 | 9.9 | 0.037 | TC | AA | AA | GG | GG |

| | | | | | | | | | |
|---|---|---|---|---|---|---|---|---|---|
| YUNONG168 | Cultivar | 54.6 | 12.9 | 0.030 | TT | CC | AG | GG | AA |
| YUNONG188 | Cultivar | 74.9 | 8.4 | 0.030 | CC | CC | AA | AG | GG |
| YUNONG612 | Cultivar | 48.1 | 8.5 | 0.049 | TT | CC | AA | AA | GG |
| YUNONG903 | Cultivar | 74.1 | 12.4 | 0.046 | NN | CC | AA | AA | GG |
| ZHENGMAI161 | Cultivar | 154.3 | 18.9 | 0.044 | CC | CC | GG | AA | GG |
| ZHENGMAI1921 | Cultivar | 51.1 | 7.0 | 0.022 | TC | CC | AA | GG | GG |
| ZHENGMAI20 | Cultivar | 79.4 | 12.3 | 0.046 | TT | CC | GG | GG | AA |
| ZHENGMAI201 | Cultivar | 66.6 | 9.9 | 0.030 | TT | CC | GG | GG | AG |
| ZHENGMAI216 | Cultivar | 128.0 | 15.2 | 0.032 | TT | CC | GG | GG | AA |
| ZHENGMAI22 | Cultivar | 109.8 | 12.5 | 0.026 | TT | CC | GG | GG | GG |
| ZHENGMAI33 | Cultivar | 149.3 | 19.6 | 0.039 | CC | CC | GG | GG | GG |
| ZHENGMAI366 | Cultivar | 77.8 | 13.5 | 0.035 | CC | AA | GG | GG | GG |
| ZHENGMAI369 | Cultivar | 64.7 | 11.3 | 0.027 | CC | CC | GG | GG | AA |
| ZHENGMAI379 | Cultivar | 32.2 | 8.0 | 0.031 | CC | TC | AA | AA | GG |
| ZHENGMAI63 | Cultivar | 83.8 | 12.6 | 0.037 | TC | CC | AA | GG | GG |
| ZHENGMAI65 | Cultivar | 91.7 | 17.2 | 0.040 | TT | CC | GG | AA | AA |
| ZHONG892 | Cultivar | 71.8 | 12.5 | 0.030 | CC | CC | AA | GG | GG |

| | | | | | | | | | |
|---|---|---|---|---|---|---|---|---|---|
| ZHONGMAI166 | Cultivar | 67.0 | 11.8 | 0.041 | NN | CC | AA | AA | AA |
| ZHONGMAI255 | Cultivar | 86.8 | 12.9 | 0.021 | CC | CC | GG | AA | AA |
| ZHONGMAI29 | Cultivar | 57.1 | 8.1 | 0.031 | CC | AA | GG | AA | GG |
| ZHONGMAI36 | Cultivar | 41.1 | 6.2 | 0.029 | TT | CC | GG | GG | GG |
| ZHONGZHIMAI16 | Cultivar | 50.3 | 12.6 | 0.037 | CC | AA | GG | AA | GG |
| ZHONGZHIMAI22 | Cultivar | 58.1 | 8.5 | 0.046 | TT | AA | GG | AA | AA |
| ZHOUMAI16 | Cultivar | 68.7 | 9.6 | 0.038 | CC | AA | GG | AA | AA |
| ZHOUMAI22 | Cultivar | 66.2 | 8.4 | 0.037 | CC | AA | AA | GG | GG |
| ZHOUMAI36 | Cultivar | 30.3 | 6.6 | 0.027 | TT | CC | GG | AA | GG |
| ZHOUMAI37 | Cultivar | 70.4 | 9.4 | 0.042 | TT | AA | AA | AA | GG |
| ZHOUMAI38 | Cultivar | 40.2 | 7.8 | 0.031 | CC | AA | AA | AA | GG |
| ZHOUMAI49 | Cultivar | 87.7 | 8.3 | 0.029 | TT | CC | GG | GG | AA |
| ZONGXINMAI998 | Cultivar | 79.1 | 12.1 | 0.033 | CC | CC | GG | AG | GG |
| WP-001 | Advanced lines | 75.1 | 8.3 | 0.036 | TT | CC | GG | GG | GG |
| WP-002 | Advanced lines | 85.0 | 10.9 | 0.035 | CC | TC | GG | GG | AA |
| WP-003 | Advanced lines | 137.5 | 13.4 | 0.030 | TC | AA | AA | GG | GG |
| WP-004 | Advanced lines | 75.7 | 13.0 | 0.028 | CC | CC | GG | GG | GG |

| | | | | | | | | | |
|---|---|---|---|---|---|---|---|---|---|
| WP-005 | Advanced lines | 73.5 | 12.8 | 0.033 | CC | CC | GG | AA | GG |
| WP-006 | Advanced lines | 123.8 | 15.7 | 0.031 | TT | TC | GG | AG | GG |
| WP-007 | Advanced lines | 69.4 | 9.8 | 0.022 | TT | TC | GG | AA | GG |
| WP-008 | Advanced lines | 60.2 | 8.3 | 0.036 | CC | AA | GG | GG | GG |
| WP-012 | Advanced lines | 130.9 | 11.9 | 0.034 | CC | AA | AA | GG | AG |
| WP-013 | Advanced lines | 73.8 | 10.1 | 0.037 | TT | AA | GG | AA | GG |
| WP-014 | Advanced lines | 154.8 | 16.5 | 0.034 | NN | CC | AG | GG | GG |
| WP-015 | Advanced lines | 141.4 | 17.7 | 0.048 | TC | CC | GG | GG | AA |
| WP-016 | Advanced lines | 123.7 | 14.3 | 0.032 | CC | AA | AG | GG | GG |
| WP-023 | Advanced lines | 86.6 | 12.6 | 0.025 | TC | CC | GG | GG | GG |
| WP-024 | Advanced lines | 70.8 | 7.9 | 0.028 | TT | CC | AA | GG | GG |
| WP-025 | Advanced lines | 66.5 | 8.9 | 0.039 | TT | AA | GG | GG | AA |
| WP-026 | Advanced lines | 54.6 | 9.2 | 0.023 | TT | CC | AA | GG | GG |
| WP-036 | Advanced lines | 87.7 | 9.3 | 0.027 | TT | CC | GG | GG | GG |
| WP-037 | Advanced lines | 102.8 | 13.1 | 0.035 | CC | AA | GG | GG | AA |
| WP-038 | Advanced lines | 143.5 | 12.7 | 0.040 | CC | CC | AA | GG | AA |

RL: root length; RA: root surface area; RV: root volume; RT: root tips; RW: root dry weight.
