# Supplementary material for: Genetic Basis of Seedling Root Traits in Common Wheat (Triticum aestivum L.) Identified by Genome-Wide Linkage Mapping
Source: Plants (Basel). 2025 Feb 6;14(3):490. doi: 10.3390/plants14030490 (PMC11820154; doi:10.3390/plants14030490)
Supplement: Supplementary file 1 [file plants-14-00490-s001.zip › Table S4.pdf]

Table S4 The primers used for the qRT-PCR of the candidate gene identified in Wp-072/Wp-119 RIL population

Candidate gene	Forward sequence (3'-5')	Reverse sequence (3'-5')
TraesCS1D01G018200	CCACGGAGATCCTCTTCCTC	GGCTCCTGGCAAACATACTG
TraesCS1D01G216500	AGAGATGCCACTCCTTGTCC	TGGAAGAGATCCGAGGAGGA
TraesCS1D01G336900	TGTCCATCCTGCACTCCAAC	GTCAGAACCTGGAGGGAGTC
TraesCS2A01G220500	TTGGTGCAATCCGTGGTATG	TCTCCTTCTCCTCCTTCCCT
TraesCS2D01G344600	TCTCGACCTCTCCAAGAACG	GAGCTTGAGGGAGTTGAGGT
TraesCS2D01G548900	AAGGCCGCCTAGAGTTTCTT	CCTCTCAGCCACATCAAAGC
TraesCS3A01G101400	GAACCCAAGAAGGCGAGAAC	CTTGCCGAGTAAACTTGCGA
TraesCS1D01G018200	CCACGGAGATCCTCTTCCTC	GGCTCCTGGCAAACATACTG
TraesCS1D01G216500	AGAGATGCCACTCCTTGTCC	TGGAAGAGATCCGAGGAGGA
